# Design and Optimization of a Novel MEMS Tuning Fork Gyroscope Microstructure

**DOI:** 10.3390/mi13020172

**Published:** 2022-01-24

**Authors:** Chuanguo Xiong, Pengjun Zeng, Weishan Lv, Fengming Lu, Ming Zhang, Yuhua Huang, Fulong Zhu

**Affiliations:** 1 School of Mechanical Science and Engineering, Huazhong University of Science and Technology, Wuhan 430074, China; chuanguo_xiong@163.com (C.X.); tiaoyuzpj@163.com (P.Z.); l1293024905@163.com (W.L.); huangyuhua@hust.edu.cn (Y.H.); 2 Quality Engineering Center, China Aero Polytechnology Establishment, Beijing 100028, China; miya_lfm@126.com (F.L.); bit_zm@163.com (M.Z.)

**Keywords:** tuning fork gyroscope, mechanical sensitivity, mode shape, transmission efficiency, Taguchi method

## Abstract

This paper presents the design and optimization of a novel MEMS tuning fork gyroscope microstructure. In order to improve the mechanical sensitivity of the gyroscope, much research has been carried out in areas such as mode matching, improving the quality factor, etc. This paper focuses on the analysis of mode shape, and effectively optimizes the decoupling structure and size of the gyroscope. In terms of structural design, the vibration performance of the proposed structure was compared with other typical structures. It was found that slotting in the middle of the base improved the transmission efficiency of Coriolis vibration, and opening arc slots between the tines reduced the working modal order and frequency. In terms of size optimization, the Taguchi method was used to optimize the relevant feature sizes of the gyroscope. Compared with the initial structure, the transmission efficiency of Coriolis vibration of the optimized gyroscope was improved by about 18%, and the working modal frequency was reduced by about 2.7 kHz. Improvement of these two indicators will further improve the mechanical sensitivity of the gyroscope.

## 1. Introduction

The application of traditional gyroscopes such as mechanical rotor gyroscopes [1,2], laser gyroscopes [3], fiber optic gyroscopes [4], etc., has been severely restricted due to their large size, high cost and unsuitability for mass production. Benefiting from the small size, low cost, low power consumption and batch fabrication, MEMS gyroscopes have been widely used in the fields of automotive safety, mobile robots, consumer electronics, aerospace navigation, military weapons, etc., over the past few decades [5,6,7,8,9]. According to different vibration structures, MEMS gyroscopes can be divided into gimbal gyroscopes, tuning fork gyroscopes, vibrating ring gyroscopes, solid-state gyroscopes, etc. [10]. Among them, the tuning fork gyroscope is mostly preferred due to its superior performance with regard to its flat structure for mass production, common-mode rejection, and low power consumption [11,12,13]. The sensitivity of the tuning fork gyroscope is one of the main challenges in terms of superior performance [14].

There are generally two ways to improve the sensitivity of tuning fork gyroscopes: peripheral circuit gain and improving mechanical sensitivity. Much research has been conducted on the former [15,16,17,18], however, it is not enough to enhance sensitivity only by circuit gain. Without sufficient mechanical sensitivity, improvements in circuit gain are not very useful. Therefore, many scholars have carried out related research, such as performing modal matching through various techniques [19,20,21,22], improving the quality factor [23,24], and suppressing the zero-rate output drift, etc. For example, Behbahan et al. proposed a systematic post fabrication technique to reduce the modal frequency differences between the *n* = 2 and *n* = 3 modes in an axisymmetric resonator [19]. Li et al. presented a leverage mechanism to improve the mechanical sensitivity of a MEMS gyroscope [14]. Xu et al. investigated a tuning fork gyroscope with a polygon-shaped vibration beam, and improved the sensitivity by optimizing the spindle azimuth [25]. Nguyen et al. proposed a z-axis tuning fork gyroscope with a freestanding and anti-phase controlled architecture, which could improve the performance by limiting the influence of air damping [11]. Wu et al. reported a z-axis quartz tuning fork gyroscope based on shear stress detection, and designed a symmetric tapered beam to increase the sensitivity [26]. Wang et al. designed the multiple-beam tuning-fork structure to achieve high quality factors, promoting the sensitivity [13]. To suppress the zero-rate output drift of MEMS tuning fork gyroscopes, Xu et al. proposed a temperature-induced stress release method [27].

This study focuses on the mode shape analysis of the gyroscope to achieve the purpose of optimizing the mechanical sensitivity. For a tuning fork gyroscope to work properly, it includes a drive structure to provide drive motion and a sensing structure to detect Coriolis vibration signals. However, the kinetic coupling between the two structures may interfere with the detection signal and reduce the mechanical sensitivity [28]. In this paper, a novel tuning fork gyroscope decoupling microstructure is proposed, which produces a relative separation between driving motion and sensing motion. Furthermore, by analyzing the mode shape of the model, the structure and size of the gyroscope are optimized, thereby improving the mechanical sensitivity. The material of the gyroscope is quartz, benefiting from good piezoelectric effect, insulation, mechanical properties and high quality factor. Details of the gyroscope structure and analysis of mode shape are provided in Section 2. Section 3 compares the advantages of the proposed structure over other typical structures and optimizes the relevant feature sizes of the gyroscope with the Taguchi method. Section 4 contains the discussion. Section 5 concludes this paper with a summary.

## 2. Materials and Methods

### 2.1. Architectural Design

**Figure 1:**
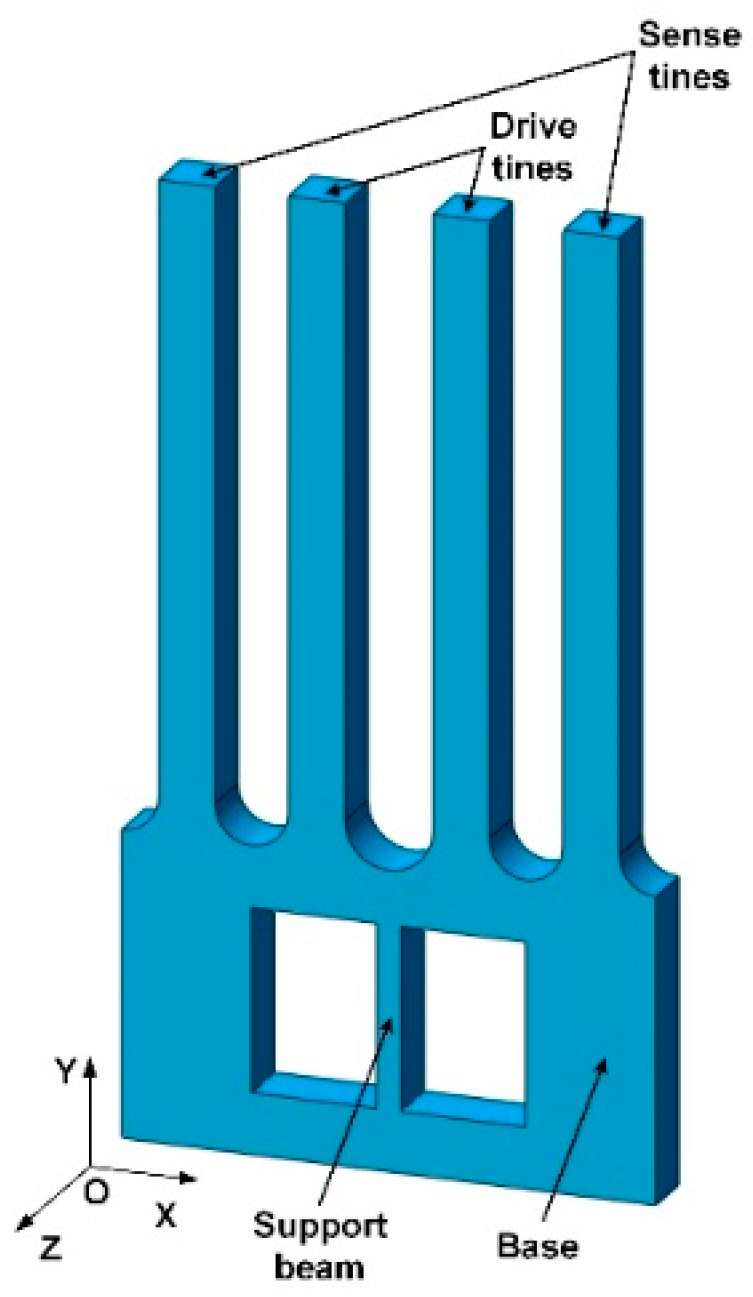
Structure of decoupling MEMS tuning fork gyroscope.

A novel MEMS tuning fork gyroscope microstructure is proposed, as shown in Figure 1. The gyroscope includes two drive tines, two sense tines, and a base with a support beam. The drive tines are used to provide driving motion, while the sense tines are used to detect the Coriolis signal. The base transmits the Coriolis force generated on the drive tines to the sense tines to eliminate the coupling between the driving motion and the sensing motion.

**Figure 2:**
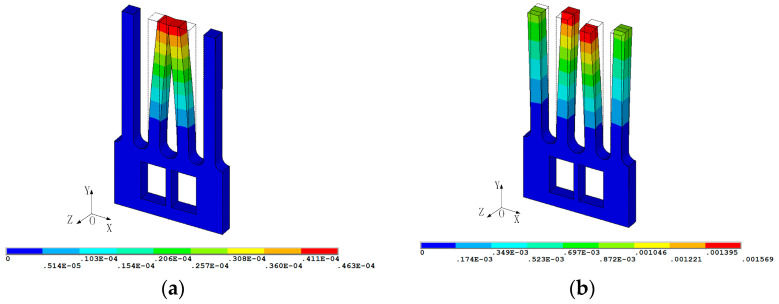
Working mode of the MEMS gyroscope: (**a**) driving mode (**b**) sensing mode.

When the gyroscope is in driving mode, as shown in Figure 2a (displacement field distribution; the unit of the analysis result is meters), the drive tines will move in opposite directions in same plane. Once there is an angular velocity input in the Y direction, the drive tines will generate Coriolis vibration in the Z direction. The Coriolis force on the two drive tines is not in the same plane, thus forming a coupling. This coupling will cause the torsion of the base, thereby transmitting the Coriolis vibration on the drive tines to the sense tines, as shown in Figure 2b. The drive tines and sense tines of the gyroscope are relatively independent, where the driving motion and sensing motion do not interfere with each other. Thus, the decoupling microstructure relatively solves the problem of interdigital motion coupling and capacitive parasitic coupling, which is conducive to signal detection.

**Figure 3:**
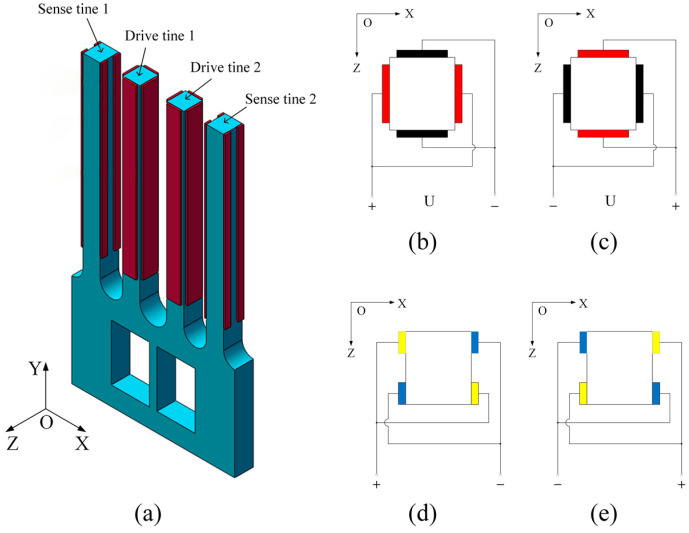
Schematic of the gyroscope electrodes arrangement: (**a**) overall electrode arrangement, (**b**) electrode directions of drive tine 1, (**c**) electrode directions of drive tine 2, (**d**) electrode directions of sense tine 1, (**e**) electrode directions of sense tine 2.

The conversion of vibration and electrical signals in the tuning fork gyroscope microstructure is based on the piezoelectric effect, thereby quartz crystals with this property are selected as a material for manufacturing. After stimulation by the driving voltage and angular velocity signal, the gyroscope outputs an electrical signal to the external circuit, and vibration characteristics are obtained by analyzing the electrical signal. The overall electrode arrangement of the gyroscope is shown in Figure 3a. Figure 3b,c represent the electrode directions of drive tine 1 and drive tine 2, and Figure 3d,e represent the electrode directions of sense tine 1 and sense tine 2, respectively. To simplify the model, since the actual electrode coating is very thin, its influence is ignored in the subsequent mode shape analysis of the gyroscope.

### 2.2. Mode Shape

The actual vibration of the system can be understood as the superposition of the various mode shapes, according to the relevant knowledge of mechanical vibration. The system response to the initial stimulus follows Formula (1). ^®^ general response of the system to any stimulus follows Formula (2) [29].

(1) $$\left\{q\left(t\right)\right\}=\sum_{r=1}^{n}\left({\left\{u^{\left(r\right)}\right\}}^{T}\left[m\right]\left\{q_{0}\right\}\cos\omega_{r}t+{\left\{u^{\left(r\right)}\right\}}^{T}\left[m\right]\left\{q_{0}^{\prime }\right\}\frac{1}{\omega_{r}}\sin\omega_{r}t\right)\left\{u^{\left(r\right)}\right\}$$

(2) $$\left\{q\left(t\right)\right\}=\sum_{r=1}^{n}\left\{u^{\left(r\right)}\right\}\frac{N_{0r}/\omega_{r}^{2}}{\sqrt{{\left[1-{\left(\omega /\omega_{r}\right)}^{2}\right]}^{2}+{\left(2\xi_{r}\omega /\omega_{r}^{2}\right)}^{2}}}\sin\left(\omega t-\varphi_{r}\right)$$

where {*q*(*t*)} is the system response, {*u^(r)^*} is the *r*-th modal vector, [*m*] is the mass matrix, {*q*_0_} is initial displacement, {*q^′^*_0_} is initial velocity, *ω_r_* is the *r*-th natural frequency, *N*_0*r*_ is excitation amplitude, ω is excitation frequency, *ξ_r_* is damping ratio, and *φ_r_* is phase of the system response.

Formula (1) shows that in the total vibration of the system, the initial motion conditions of the system determine the degree to which the *r*-th mode is excited. If {*q*_0_} approximates the *s*-th modal vector, the system will vibrate almost according to the *s*-th mode. Formula (2) shows that the amplitude of the system response is related to the excitation frequency. Obviously, when we apply excitation to make the gyroscope work in the driving mode while the excitation frequency is equal to the driving modal frequency, the gyroscope will resonate in the driving mode. At this time, it is uncertain whether the sensing vibration aroused by the angular velocity vibrates in the manner of the sensing mode.

**Figure 4:**
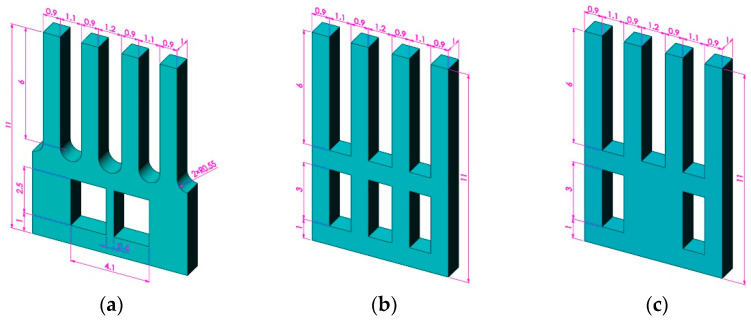
Model of the mode test: (**a**) type A, (**b**) type B, and (**c**) type C.

**Figure 5:**
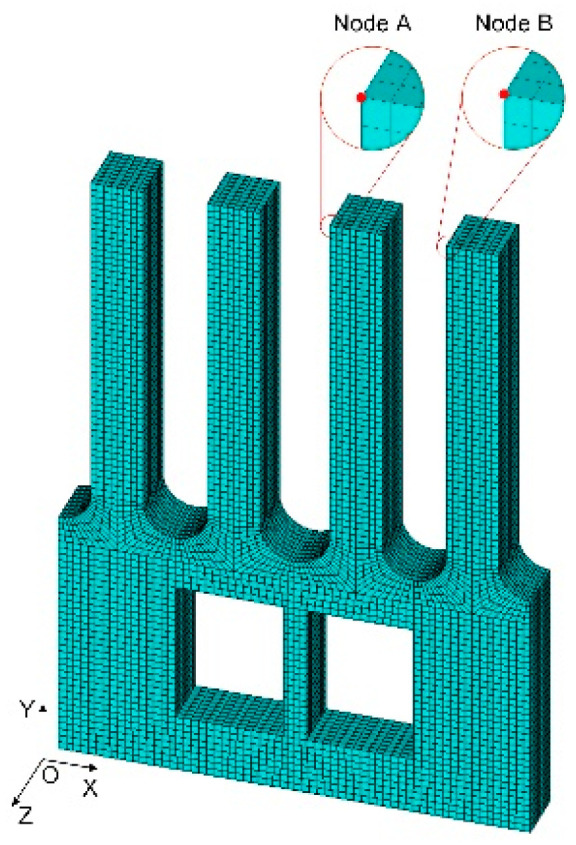
Mesh model of type A.

**Figure 6:**
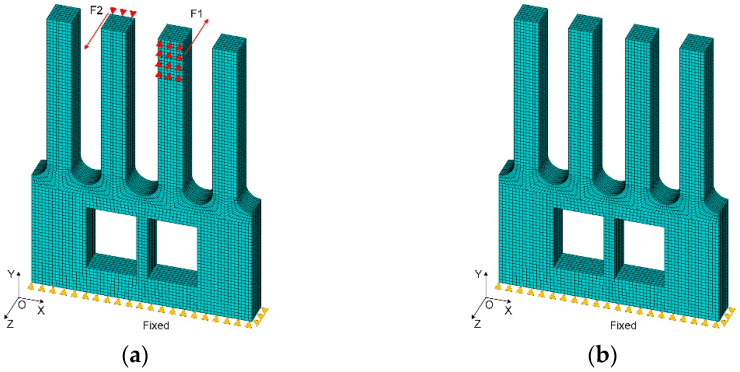
Load of the finite element model: (**a**) harmonic analysis, and (**b**) modal analysis.

To verify the conjecture, three sets of analyses have been conducted, and the analysis models of the gyroscope are shown in Figure 4. Dimensions of all models are in millimeters, and are analyzed by Ansys software. Moreover, the geometric structure of the designed gyroscope is simple and symmetrical, so it is more suitable to select hexahedral elements for meshing. Taking type A as an example, its mesh model is shown in Figure 5. The node A coincides with the endpoint of the right drive tine, and the node B coincides with the endpoint of the right sense tine. Their specific locations are indicated in Figure 5. The analysis index is the vibration state of node A and node B in the actual sensing vibration and sensing mode, and analyzing the sensing vibration with harmonic analysis. In addition, a zero-displacement constraint is applied to the bottom of the gyroscope, and a set of sinusoidal forces with equal amplitude and opposite phase are applied to the front and back surface of the drive tines, respectively. Moreover, the amplitude of the load *F* is 0.0001 N, while the frequency of it is 200 Hz less than the sensing modal frequency. The application area is 1/3 of the upper part of the drive tine. The loading diagram is shown in Figure 6a. In the sensing mode, a zero-displacement constraint of the bottom is the only load, which is shown in Figure 6b.

## 3. Results and Analysis

The actual sensing vibration state is in line with the sensing mode. As shown in Table 1, in the harmonic analysis, the amplitude ratio and phase difference of the displacement between node A and node B are both approximate to the sensing mode. This shows that although the vibration frequency is different from the sensing modal frequency by 200 Hz, when the drive tines vibrate in the Z direction oppositely affected by Coriolis force, the sense tines will also vibrate in the Z direction correspondingly, and the vibration state is almost consistent with the sensing mode. According to Formula (1), at this time, the Coriolis vibration state of the gyroscope is almost completely determined by the sensing mode. Therefore, it is credible to evaluate the transmission of Coriolis vibration by use of the sensing mode, which can greatly reduce the calculation required.

The gyroscope generally works in resonance, and it is known from Table 1 that the displacement of the drive tines affected by Coriolis force has little connection with the shape of the base. Hence, the key to improving the mechanical sensitivity of the gyroscope is in improving the transmission efficiency of the Coriolis signal from the drive tines to the sense tines as much as possible. The transmission efficiency is denoted as *ŋ* and is defined by the formula given below.

(3) η=ZBZA

where *Z_B_* is the displacement of node *B* in the sensing mode, and *Z_A_* is the displacement of node *A*.

### 3.1. Structural Analysis

The mechanical sensitivity of the gyroscope is denoted as S and can be calculated according to Formula (4), where $Y_{0}$ is sense tine amplitude, Ω is angular velocity, *η* is transmission efficiency of the Coriolis vibration, *F* is driving force, and the driving direction quality factor and resonant frequency are represented by $Q_{x}$ and $\omega_{x}$, respectively. In addition, m is mass of the drive tine, and $Q_{y}$ and$\;\omega_{y}$ represent the quality factor and resonant frequency of driving direction, respectively.

(4) $$S=\frac{Y_{0}}{\Omega }=\frac{2\eta FQ_{x}}{m\omega_{x}\omega_{y}^{2}}\cdot \frac{1}{\sqrt{{\left(1-{\left(\frac{\omega_{x}}{\omega_{y}}\right)}^{2}\right)}^{2}+{\left(\frac{\omega_{x}}{\omega_{y}Q_{y}}\right)}^{2}}}$$

According to the calculation formula of mechanical sensitivity, the gyroscope microstructure can be optimized from the following aspects:

(1) The transmission efficiency of Coriolis vibration *ŋ* should be as high as possible;
(2) The working modal (driving mode and sensing mode) order and frequency should be as low as possible, which can achieve greater response amplitude in the structural vibration. When the excitation frequency is close to the *r*-th natural frequency *ω_r_*, the system response {*q*(*t*)} is approximately expressed by the following formula.
  (5) $$\left\{q\left(t\right)\right\}\approx \left\{u^{\left(r\right)}\right\}\frac{N_{0r}}{2\xi_{r}\omega_{r}}\sin\left(\omega_{r}t-\frac{\pi }{2}\right)$$
  where {*u^(r)^*} is the *r*-th modal vector, *N*_0*r*_ is excitation amplitude, and *ξ_r_* is damping ratio.

It is known from Formula (5) that the lower the working modal frequency is, the greater the response amplitude is, and therefore, the higher is the sensitivity of the gyroscope;

(3) The driving mode and the sensing mode should be adjacent to reduce the effects of interfering modes.

**Figure 7:**
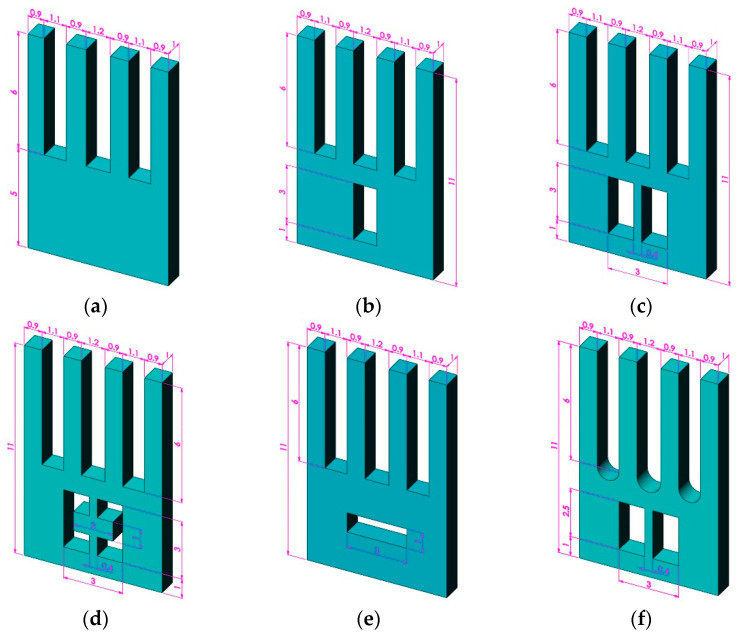
Six other typical models of the gyroscope: (**a**) type D: initial structure, (**b**) type E: single hole in the base, (**c**) type F: double hole in the base, (**d**) type G: spindle beam between two holes, (**e**) type H: single hole in horizontal direction, and (**f**) type I: opening arc slots between the tines.

In addition to the three structures shown in Figure 4, six typical structures are analyzed, which are shown in Figure 7. The mode frequency and Coriolis vibration transmission efficiency results of all models are shown in Table 2.

**Figure 8:**
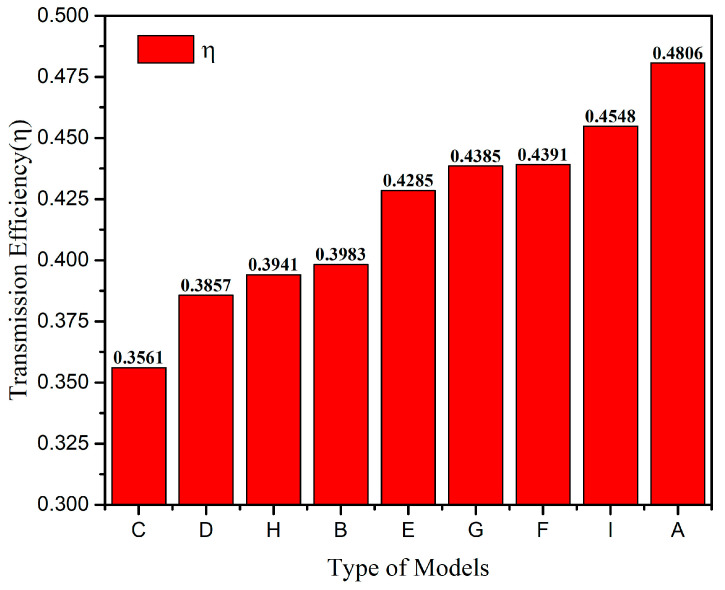
Bar chart of the transmission efficiency *ŋ* of various typical structures.

As seen from Figure 8, slot structure types have different effects on the transmission efficiency. Based on type D, slotting on the sides of the base will reduce the transmission efficiency, such as in type C. On the contrary, slotting in the middle of the base will improve the transmission efficiency, such as in types E, F and G. According to Table 1, opening arc slots between the tines can reduce the working modal order and frequency, such as in type I. Therefore, combining the two advantageous features—slotting in the middle of the base and opening arc slots between the tines—can greatly improve the mechanical sensitivity of the gyroscope. The proposed type A not only has the above characteristics, but additionally, its base protrudes slightly outward, so that the sense tine is also symmetrical in a local area, thereby obtaining better vibration performance.

After modal analysis of different models, it was found that type A, with high symmetry, had the best vibration performance. Its transmission efficiency was the highest, and the working mode order and frequency were also the lowest. In addition, the driving and sensing modes were adjacent to each other. In summation, type A perfectly meets the three preset performance requirements.

### 3.2. Size Optimization with Taguchi Method

**Figure 9:**
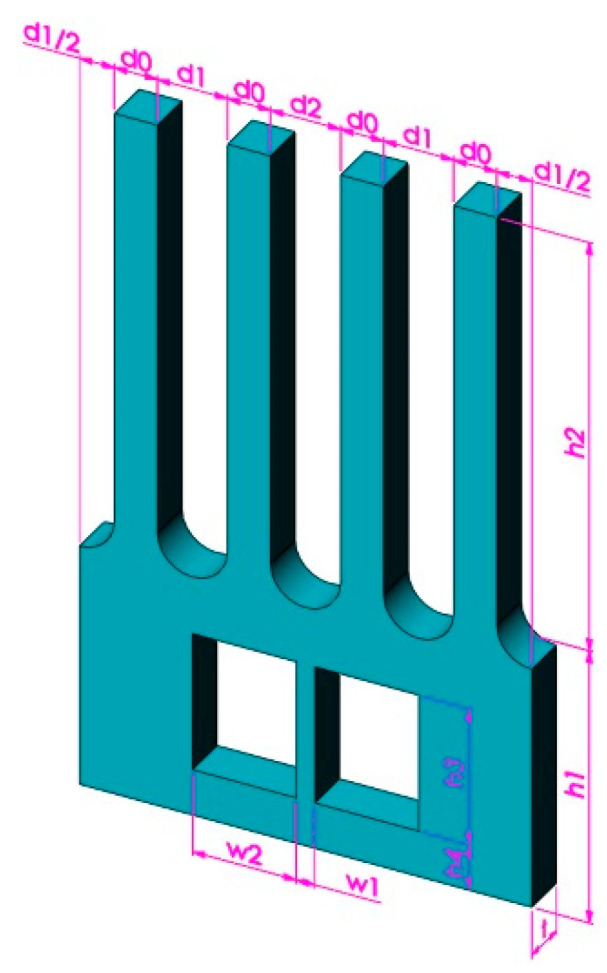
Feature size codes of the gyroscope.

The Taguchi method is a reliable design method that guides the optimal design of products in complex environments. In this section, the Taguchi method is used to optimize the relevant feature sizes of the gyroscope. The feature size codes of the gyroscope are shown in Figure 9. The height of tines *h*2, drive-sense interval *d*1, drive–drive interval *d*2, width of the support beam *w*1, and height of the groove *h*3, are the signal factors. The noise factor is the mesh size of the finite element model. Parameters and their levels are given in Table 3 and Table 4.

The best experimental result is evaluated by the calculation of signal to noise ratio (SNR) in the Taguchi method. The larger the SNR is, the smaller the quality loss and the better the product quality. To obtain the optimum result, three basic categories which are smaller-the-better, larger-the-better and nominal-the-best characteristics are calculated by the formulas given below [30].

(a) Smaller-the-better characteristic:
  (6) $$\mathrm{SNR}=-10\mathrm{lg}\left(\frac{1}{n}\sum_{i=1}^{n}y_{i}^{2}\right)$$
(b) Larger-the-better characteristic:
  (7) $$\mathrm{SNR}=-10\mathrm{lg}\left(\frac{1}{n}\sum_{i=1}^{n}\frac{1}{y_{i}^{2}}\right)$$
(c) Nominal-the-best characteristic:
  (8) $$\mathrm{SNR}=-10\mathrm{lg}\left(\frac{1}{n}\sum_{i=1}^{n}{\left(y_{i}-m\right)}^{2}\right)$$
  where *y_i_* is measured values, and *n* and *m* are number of experiments and nominal value respectively.

The orthogonal experiment table was created according to Table 3 and Table 4, and the corresponding finite element model was established to analyze according to different combinations of each factor level. The observed experiment results include the following items:

(1) The transmission efficiency of Coriolis vibration which meets the larger-the-better characteristic;
(2) Frequency difference between the driving mode and sensing mode. When the frequency difference between the two is large, the working bandwidth of the gyroscope meets Formula (9) [31]:
  (9) BW=0.54ΔF

where Δ*F* is the frequency difference between the driving mode and sensing mode.

Given that the applications of tactical and inertial gyroscope require bandwidths of around 100 Hz [9], the frequency difference Δ*F* should be maintained at about 200 Hz. For the convenience of calculation, this paper defines the difference between Δ*F* and 200 Hz as a new variable Δ*f*, which meets the smaller-the-better characteristic and follows the Formula (10) given below:

(10) Δf=|ΔF−200|

(3) The driving modal frequency which meets the smaller-the-better characteristic;
(4) The drive coupling coefficient which meets the smaller-the-better characteristic. Similar to Section 3.2, the drive coupling coefficient is denoted as *λ* and defined by the Formula (11) given below.
  (11) λ=XBXA
  where *X_B_* is the displacement of node *B* in the driving mode, and *X_A_* is the displacement of node *A*.

Since it is preferable for the undesired coupling motion between the drive tines and the sense tines to be separated, it is better to keep the sense tines stationary when the drive tines are vibrating in the opposite direction, and, therefore, the drive coupling coefficient needs to be as low as possible.

**Figure 10:**
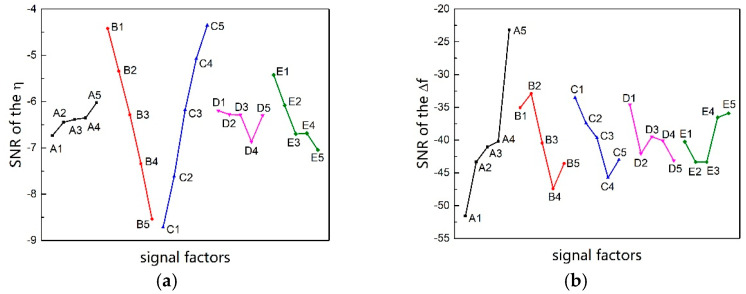

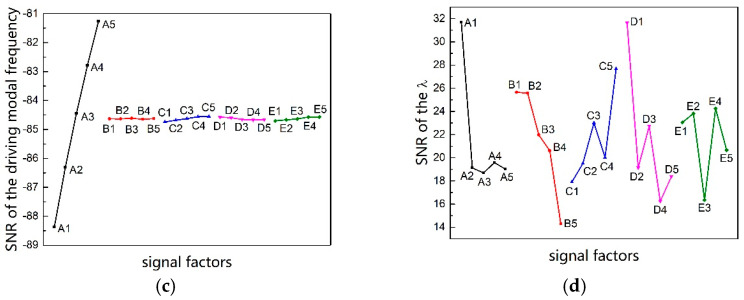
SNR effect diagram of different result index: (**a**) SNR effect diagram of the transmission efficiency, (**b**) SNR effect diagram of the frequency difference, (**c**) SNR effect diagram of the driving modal frequency, and (**d**) SNR effect diagram of the drive coupling coefficient.

**Figure 11:**
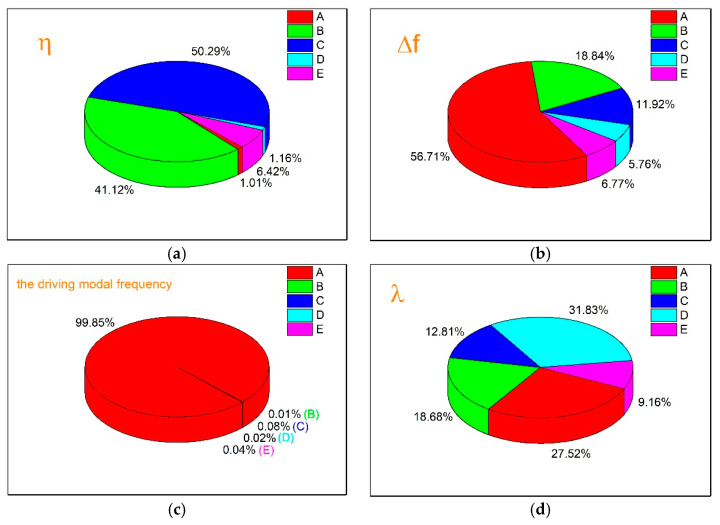
The contribution rate of each signal factor to the SNR of different outcome indicators: (**a**) the contribution ratio of each signal factor to the SNR of the *ŋ*, (**b**) the contribution ratio of each signal factor to the SNR of the Δ*f*, (**c**) the contribution ratio of each signal factor to the SNR of the driving modal frequency, and (**d**) the contribution ratio of each signal factor to the SNR of the *λ*.

According to the different quality characteristics of each result index, the corresponding SNR calculation formula is used for data processing. The relationship between the SNR of different result indexes and the signal factors is shown in Figure 10. Moreover, variance analysis is performed to examine the influence degree of each factor on different outcome indicators, as shown in Figure 11.

The influence degree of every signal factor on each outcome indicator is different. As can be seen from Figure 11, for the indicator (a), the contribution rate of each signal factor is ranked as C>B≫E>D≈A, where the effect of factor B and factor C is huge. For indicator (b), the contribution rate of each signal factor is ranked as A>B>C>E>D, where the effect of factor A is powerful. For the indicator (c), the contribution rate of each signal factor is ranked as A≫(B≈C≈D≈E), where the effect of factor A is significant. For the indicator (d), the contribution rate of each signal factor is ranked as D>A>B>C>E, where the influence degree of each factor is little different.

In the Taguchi method, the factor that has a significant impact is prioritized, and the factor with insignificant effect is comprehensively considered according to actual needs. According to Figure 10a, the levels of factor B and factor C are selected as B1 and C5, respectively. According to Figure 10b,c, the level of factor A is selected as A5. The levels of factor D and factor E are selected as D1 and E4, taking Figure 10a–d into account. Therefore, when considering only the above four performance indicators, the optimal parameter according to the Taguchi method is A5B1C5D1E4. However, the performance indicators that need to be considered in practical application also include miniaturization, impact resistance, processing difficulty, etc. Therefore, it is necessary to further optimize the results obtained by the Taguchi method in accordance with the gyroscope application requirements.

It was found that the modal frequency will be too concentrated when the height of tines *h*2 is too high. In order to isolate the influence of the interference mode, the level of factor A was optimized from A5 to A4. Combined with the specific structure of the gyroscope, the difference between factor B and factor C cannot be too large, otherwise the two sides of the drive tine will not be sufficiently symmetrical, which will affect the gyroscope mode shape. As known from Figure 10a, within the appropriate range, a smaller value of factor B and bigger value of factor C will result in better vibration performance. Considering miniaturization and the index, the difference between factor B and factor C cannot be too large, therefore, the level of factor B was selected as B1, and the value of factor C was selected as 0.72 mm after further calculation, which was near C2 and was denoted as C2′. Considering the processing difficulty, the level of the factor D was optimized from D1 to D3. Considering the miniaturization, the level of the factor E was optimized from E4 to E2. Therefore, combined with practical application requirements, the further optimization result based on the Taguchi method is A4B1C2′D3E2.

**Figure 12:**
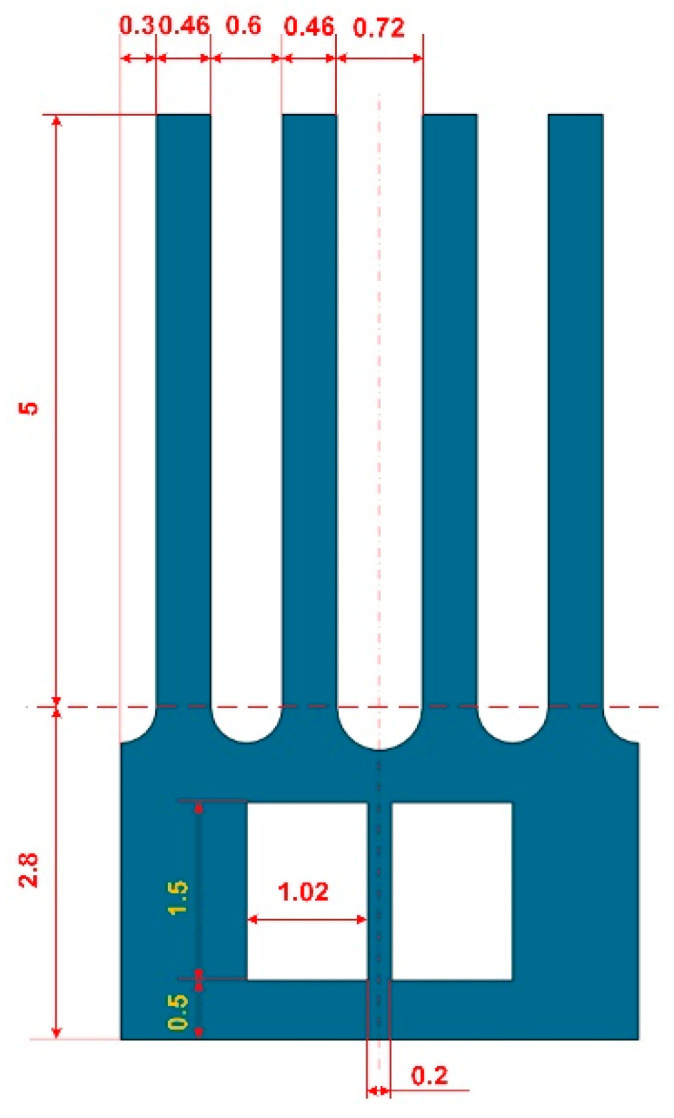
The feature sizes of the optimized gyroscope.

The feature sizes of the gyroscope optimized by the Taguchi method are shown in Figure 12, where the gyroscopic thickness is 0.5 mm.

The feature sizes of the gyroscope before and after Taguchi optimization are shown in Table 5, where the relevant size codes have been shown in Figure 9. The related vibration performance is shown in Table 6.

As can be seen from Table 6, compared with the initial structure, the transmission efficiency *ŋ* of the optimized gyroscope has improved by about 18%, and the working modal frequency has been reduced by about 2.7 kHz. According to the previous analysis, the improvement of these two indicators will further improve the mechanical sensitivity of the gyroscope.

## 4. Discussion

According to the simulation results, it was found that slotting in the middle of the base will improve the transmission efficiency of Coriolis vibration, while slotting on the sides of the base will reduce it. In addition, the transmission efficiency was improved by decreasing drive–sense interval *d*1 and increasing drive–drive interval *d*2. We believe that this may be due to the following reasons:

(1) Slotting on both sides of the base will increase the local torsional stiffness of the edge area of the base, which is not conducive to the transmission of Coriolis vibration. Slotting in the middle of the base will increase the local torsional stiffness of the middle area of the base, thereby hindering the loss of Coriolis vibration towards the middle direction, and playing a guiding and promoting role in the transmission of Coriolis vibration;
(2) The decrease in drive–sense interval *d*1 reduces the distance between the drive tine and the sense tine, thereby reducing energy loss in the transmission process and improving the transmission efficiency;
(3) The increase in drive–drive interval *d*2 increases the width of the base, thereby reducing the overall torsional stiffness of the base and facilitating the transmission of Coriolis vibration.

In terms of manufacturing, some adjustments were made to the structure and size optimization process of the gyroscope according to manufacturing process issues. For example, the hole pattern of the gyroscope base was as simple and symmetrical as possible, and the process difficulty was comprehensively considered when optimizing the size. In future studies, these considerations will benefit the fabrication and testing of the proposed quartz tuning fork gyroscope.

## 5. Conclusions

This paper designed a novel MEMS tuning fork gyroscope microstructure and optimized it from the perspective of mode shape, providing a new idea for the design of MEMS gyroscopes. Firstly, the paper demonstrated the correctness for using the mode shape to reflect the vibration state of the gyroscope. Then the finite element method was utilized to compare the vibration performance of the proposed structure with other typical structures, and the advantages of the designed structure were noted. Finally, the Taguchi method was used to optimize the relevant feature sizes of the gyroscope. In short, the research conclusions can be summarized as follows:

(1) This gyroscope microstructure worked by transmitting vibrations between the drive tine and sense tine, relatively eliminating the kinematic coupling. It was credible to evaluate the transmission efficiency of Coriolis vibration by using the sensing mode, which can greatly reduce the amount of calculation required;
(2) During gyroscope structural analysis, slotting in the middle of the base improved the transmission efficiency, and opening arc slots between the tines reduced the working modal order and frequency. Moreover, the base protruding slightly outward improved local symmetry to obtain better vibration performance;
(3) The height of tines had a large influence on the frequency difference of working mode and the frequency of driving mode, while the tine interval had a large effect on the transmission efficiency of Coriolis vibration. Optimized by the Taguchi method, the transmission efficiency was improved by about 18%, and the working modal frequency was reduced by about 2.7 kHz. The improvement of these two indicators will further improve the mechanical sensitivity of the gyroscope microstructure.

## Figures and Tables

**Table 1:** The finite element results of the mode shape.

Type	Harmonic Analysis						Modal Analysis	
	Amplitude A (μm)	Amplitude B (μm)	Phase A (°)	Phase B (°)	A_B_/A_A_	Δφ (°)	A_B_/A_A_	Δφ (°)
A	24.1411	11.8684	−0.622	179.407	0.4916	180.029	0.4806	180
B	23.0454	9.2162	−0.750	179.273	0.3999	180.023	0.3983	180
C	23.6177	8.4889	−0.765	179.260	0.3594	180.025	0.3561	180

**Table 2:** Vibration performance of different typical structures (# represents driving mode frequency, * represents sensing mode frequency).

Type	Modal Frequency (Hz)				*ŋ*
	Fifth Order	Sixth Order	Seventh Order	Eighth Order	
A	17,302.9 (#)	17,369.6 (*)	17,713.9	--	0.4806
B	--	18,460.3	18,658.5 (#)	19,475.1 (*)	0.3983
C	--	18,676.9	19,198.2 (#)	19,639.6 (*)	0.3561
D	--	19,343.1	19,384.1 (#)	19,777.7 (*)	0.3857
E	--	19,106.6 (#)	19,264.8	19,669.4 (*)	0.4285
F	--	18,565.9 (#)	19,171.6	19,436.1 (*)	0.4391
G	--	18,598.8 (#)	19,172.7	19,436.7 (*)	0.4385
H	--	19,186.2 (#)	19,305.6	19,762.5 (*)	0.3941
I	--	17,394.5 (#)	17,460.5 (*)	17,961.3	0.4548

**Table 3:** Levels of the signal factors.

Levels	A	B	C	D	E
	*h*2 (mm)	*d*1 (mm)	*d*2 (mm)	*w*1 (mm)	*h*3 (mm)
1	3.5	0.6	0.66	0.12	1
2	4	0.7	0.76	0.16	1.5
3	4.5	0.8	0.86	0.2	2
4	5	0.9	0.96	0.24	2.5
5	5.5	1	1.06	0.28	3

**Table 4:** Levels of the noise factor.

Levels	F
	Mesh Size (mm)
1	0.06
2	0.08

**Table 5:** The feature sizes of the gyroscope before and after Taguchi optimization.

Feature Sizes	*d*0	*d*1	*d*2	*h*1	*h*2	*t*	*h*3	*h*4	*w*1	*w*2
Initial Value (mm)	0.46	0.8	0.86	3.5	4.5	0.5	2	0.5	0.2	1.19
Optimized Value (mm)	0.46	0.6	0.72	2.8	5	0.5	1.5	0.5	0.2	1.02

**Table 6:** The vibration performance of the gyroscope before and after Taguchi optimization.

	Drive Modal Order	Sense Modal Order	Drive Modal Frequency (Hz)	Sense Modal Frequency (Hz)	Frequency Difference Δ*F* (Hz)	The Transmission Efficiency *ŋ*
Initial	6	5	16,832.9	16,623.2	209.7	0.4632
Optimized	5	6	13,907.8	14,101.5	193.7	0.5466
